# The clinical characteristics differences between IVF twins and naturally conceived twins with preterm infants

**DOI:** 10.1097/MD.0000000000041884

**Published:** 2025-03-21

**Authors:** Chunyan Guo, Shasha Li, Jingcai Wang, Yanqiu Wu

**Affiliations:** a Department of Physiology, Chengde Medical University, Chengde, China; b Department of Neonatology, Affiliated Hospital of Chengde Medical University, Chengde, China.

**Keywords:** clinical characteristics, in vitro fertilization, perinatal diseases, twin preterm birth

## Abstract

With the rapid development of Assisted Reproductive Technology, in vitro fertilization (IVF) has become one of the most important treatments for infertility. However, the widespread use of IVF has significantly increased the incidence of twin pregnancies, which in turn raises the risk of preterm twin births. Twin pregnancies are inherently high-risk, often associated with complications such as preterm labor, fetal growth restriction, and congenital malformations, all of which can severely impact the health of both mothers and infants. The aim of this study is to investigate the differences between IVF twin preterm infants and naturally conceived twin preterm infants. In this study, we included 144 cases of twin preterm infants born in the obstetrics department of our hospital and subsequently transferred to the neonatal unit between January 2021 and June 2024. Using a retrospective cohort design, we divided the cases into 2 groups: 72 IVF–embryo transfer cases (observation group) and 72 naturally conceived cases (control group). We compared the 2 groups in terms of general characteristics, mode of delivery, premature rupture of membranes, congenital heart disease, intracranial hemorrhage, neonatal conditions, and hospitalization to assess the impact of IVF on twin preterm infants and maternal perinatal diseases. The results showed that the observation group had a significantly higher proportion of assisted perineal lateral incisions, a higher incidence and longer duration of premature rupture of membranes (*P* < .05), and a higher incidence of atrial septal defect (*P* < .05) compared to the control group. Additionally, the incidence of neonatal hyperbilirubinemia, intracranial hypertension syndrome, and hypocalcemia was significantly higher in the observation group (*P* < .05), whereas the incidence of twin–twin transfusion syndrome was significantly lower (*P* < .05). During hospitalization, the observation group required enteral and intravenous nutrition for a significantly longer period than the control group (*P* < .05). Regarding maternal characteristics, the observation group had a higher maternal age, a greater proportion of primigravida, and significantly higher rates of hypothyroidism during pregnancy and antenatal antibiotic use compared to the control group (*P* < .05). In conclusion, IVF twin preterm infants exhibited significant differences in clinical characteristics and maternal perinatal disorders when compared to naturally conceived twin preterm infants. This suggests that IVF technology may present additional clinical management challenges while enabling twin pregnancies.

## 1. Introduction

With the rapid development of Assisted Reproductive Technology, in vitro fertilization (IVF) has become one of the most important methods for addressing infertility.^[1]^ In recent years, the widespread use of IVF technology has significantly increased the incidence of twin pregnancies. Statistics show that the rate of twin pregnancies in IVF pregnancies is notably higher compared to naturally conceived pregnancies, reaching approximately 30%, compared to around 1% in naturally conceived women.^[2]^ This increase in twin pregnancies is largely attributed to the growing use of IVF in infertility treatment.^[2]^ Consequently, the rate of preterm births in IVF twin pregnancies has risen, with preterm birth rates as high as 50% in IVF twin pregnancies, significantly surpassing those in naturally conceived twin pregnancies.^[3]^

Twin pregnancies are inherently high-risk, with significantly higher rates of complications such as preterm birth, fetal growth restriction, and congenital anomalies compared to singleton pregnancies.^[4]^ Preterm infants, especially very preterm (<28 weeks gestational age) and early preterm infants (28–32 weeks gestational age), face numerous serious health issues, including respiratory distress syndrome, intraventricular hemorrhage, and sepsis.^[5]^ Although advances in neonatal intensive care technology have greatly improved the survival rates and quality of life for preterm infants, their short- and long-term health problems remain significant.^[6]^

While IVF has been instrumental in facilitating twin pregnancies, there is a lack of systematic research regarding whether twin preterm infants born via IVF differ significantly from naturally conceived twin preterm infants in terms of clinical characteristics, prognosis, and management strategies. Some studies suggest that IVF twin pregnancies may differ from naturally conceived twin pregnancies in terms of gestational characteristics, birth weight, and length of hospital stay.^[7]^ These differences may result from factors such as the IVF technique itself, the maternal health status, embryo selection methods, or other related variables.^[8,9]^

Research has generally focused on the overall health outcomes of IVF singleton or multiple pregnancies, with limited comparative analyses of IVF twin preterm infants versus naturally conceived twin preterm infants.^[10]^ Previous studies have identified differences in the overall health outcomes of IVF twin pregnancies.^[10]^ These studies suggest that IVF twin preterm infants may exhibit significant differences in birth weight and physiological parameters at birth, though comparisons of long-term development and health outcomes remain unclear.^[11]^ Additionally, variations in IVF techniques (e.g., IVF–intracytoplasmic sperm injection, frozen embryo transfer, etc) may influence the clinical characteristics of twin preterm infants, but relevant research in this area is insufficient.^[12]^

The aim of this study is to investigate the differences between IVF twin preterm infants and naturally conceived twin preterm infants in terms of gestational management, birth weight, preterm complication rates, and prognosis, by comparing their clinical characteristics. Through a retrospective analysis of clinical data, this study aims to provide a reference for clinical practice, improve the quality of care and prognosis for twin preterm infants, and offer foundational data for future research in this area.

## 2. Methodology

### 2.1. Research design

This study was approved by the Ethics Committee of the Affiliated Hospital of Chengde Medical University. This study was designed as a retrospective cohort study with the aim of comparing the clinical characteristics of IVF twin preterm infants to those of naturally conceived twin preterm infants during the neonatal period and investigating the differences in maternal perinatal diseases. The goal is to provide a scientific basis for the clinical management of IVF twin preterm infants. The study period spanned from January 2021 to June 2024, with data obtained from the hospital’s existing electronic medical record system. The collected data included: general information of the study subjects (e.g., gestational age, mode of delivery); maternal perinatal conditions (e.g., gestational hypertension, premature rupture of membranes, and placenta previa); neonatal conditions (e.g., neonatal asphyxia, neonatal pneumonia, meconium aspiration syndrome, and bronchopulmonary dysplasia); and neonatal interventions (e.g., pulmonary surfactant use, respiratory support). The study was approved by the hospital’s ethics committee and adhered to privacy protection standards. All data were anonymized and de-identified to ensure confidentiality and were used solely for academic publication purposes.

### 2.2. Study subjects

The study subjects were IVF twin preterm infants born in the obstetrics department of our hospital and subsequently transferred to the neonatal unit after conception via in vitro fertilization–embryo transfer during the study period (observation group). Naturally conceived twin preterm infants, born in the same department and transferred to the neonatal unit within the same timeframe, were selected as the control group. Based on inclusion and exclusion criteria, a total of 144 patients were included, with 72 cases in each group (see Table 1 for details).

**Table 1:** Comparison of general data between 2 groups of preterm infants (N/%, *X* ± SD, M).

Variable	Observation group (N = 72)	Control group (N = 72)	*Z*/*t*/*χ*²	*P* value
Gender			0.694	.408
Male	39 (54.17%)	34 (47.22%)		
Female	33 (45.83%)	38 (52.78%)		
Gestational age (wk)	34.29 ± 2.06	34.52 ± 2.32	‐0.630	.530
Birth weight (g)	1924.15 ± 418.26	1937.52 ± 494.18	‐0.175	.860
**Mode of delivery**			7.170	**.046**
Vaginal delivery	21 (29.17%)	25 (34.72%)		
Cesarean section	37 (51.39%)	44 (61.11%)		
Episiotomy-assisted delivery	14 (19.44%)	3 (4.17%)		
Gestational age–weight relationship			1.184	.550
SGA	20 (27.78%)	22 (30.56%)		
AGA	52 (72.22%)	49 (68.06%)		
LGA	0 (0.00%)	1 (1.39%)		
Amniotic fluid abnormalities	4 (5.56%)	4 (5.56%)	0.000	1.000
Placental abnormalities			3.434	.063
Placenta previa	6 (8.33%)	13 (18.06%)		
Placental abruption	1 (1.39%)	2 (2.78%)		
**PPROM**	28 (38.89%)	15 (20.83%)	6.600	**.010**
**PPROM duration (h**)	107.58 ± 183.25	51.43 ± 84.98	2.360	**.018**
APGAR score				
1 min	7.73 ± 1.64	7.82 ± 1.49	−0.345	.730
5 min	9.40 ± 1.03	9.32 ± 1.27	0.415	.680
Umbilical artery blood gas				
pH	7.22 (7.13, 7.31)	7.35 (7.29, 7.39)	0.100	.100
BE	−6.70 (−10.33, 3.03)	−4.48 (−5.63, −3.12)	0.200	.200
Severe hypoxia	1 (1.39%)	2 (2.79%)	0.000	.792

Bold values indicate statistical significance.

AGA = appropriate for gestational age, APGAR = Appearance, Pulse, Grimace, Activity, Respiration, BE = base excess, LGA = large for gestational age, PPROM = premature rupture of membranes, SGA = small for gestational age.

Inclusion criteria: The study subjects were selected based on the following inclusion criteria: (1) preterm twins born through IVF, (2) gestational age between 28 and 36 weeks, and (3) availability of complete neonatal clinical records. Exclusion criteria included: (1) twins with congenital malformations or chromosomal abnormalities, (2) maternal history of severe chronic diseases (e.g., diabetes, hypertension, or autoimmune disorders) that could significantly impact neonatal outcomes, and (3) neonates with severe perinatal complications such as intraventricular hemorrhage (IVH) grade III/IV or necrotizing enterocolitis requiring surgical intervention. These criteria were implemented to minimize the influence of early or specific diseases and other potential confounding factors on the study outcomes.

### 2.3. Diagnostic criteria

The diagnostic criteria for neonatal diseases (e.g., neonatal respiratory distress syndrome, neonatal wet lung, neonatal hypocalcemia, and neonatal hypothyroidism) were based on the relevant clinical guidelines. Similarly, the diagnosis of maternal conditions (e.g., gestational hypertension, gestational diabetes, and gestational hypothyroidism) followed strict clinical guidelines to ensure scientific validity and consistency.

### 2.4. Statistical analysis

Statistical analysis was performed using SPSS 25.0 software. Measurement data were presented as mean ± standard deviation (*X* ± SD) or median (M), with *t* tests used for data following a normal distribution and nonparametric tests applied for data that did not follow a normal distribution. Categorical data were expressed as frequency (n) and percentage (%), and the chi-square test was used for comparison. A *P*-value of <.05 was considered statistically significant.

## 3. Results

### 3.1. Comparison of general information of twin preterm infants in the 2 groups

There was no significant difference in gender distribution between the observation and control groups (*P* = .408), with 54.17% of male infants in the observation group and 47.22% in the control group; 45.83% and 52.78% of female infants, respectively. The gestational age (34.29 ± 2.06 weeks in the observation group vs 34.52 ± 2.32 weeks in the control group, *P* = .530) and birth weight (1924.15 ± 418.26 g in the observation group vs 1937.52 ± 494.18 g in the control group, *P* = .860) did not differ significantly between the 2 groups. Regarding the mode of delivery, the proportion of assisted delivery with a perineal lateral incision was significantly higher in the observation group compared to the control group (19.44% vs 4.17%, *χ*² = 7.170, *P* = .046). The proportion of cesarean section was higher in the observation group, but this difference was not statistically significant (51.39% vs 61.11%, *P* > .05). Premature rupture of membranes (PPROM) occurred more frequently in the observation group than in the control group (38.89% vs 20.83%, *P* = .010) and lasted longer (107.58 ± 183.25 hours vs 51.43 ± 84.98 hours, *P* = .018). There were no significant differences between the 2 groups in terms of the gestational age–birth weight relationship (*P* = .550). Additionally, neonatal Appearance, Pulse, Grimace, Activity, Respiration scores and umbilical artery blood gas analysis indices (pH and BE) did not differ significantly between the 2 groups, nor did the incidence of severe hypoxia (*P* = .792) (Table 1).

### 3.2. Comparison of the number of cases and birth weight of twin preterm infants in different gestational age groups

No significant differences were found in the number of cases or birth weight of twin preterm infants across different gestational age groups (27–31^+6^ weeks, 32–33^+6^ weeks, and 34–36^+6^ weeks) between the observation and control groups (*P* > .05). Specifically, at 27–31^+6^ weeks, the number of cases was 10 (13.89%) in the observation group and 10 (13.89%) in the control group; at 32–33^+6^ weeks, 19 (26.39%) cases in the observation group and 13 (18.06%) in the control group; and at 34–36^+6^ weeks, 43 (59.72%) cases in the observation group and 49 (68.06%) in the control group. The birth weights of the infants in each gestational age group were also similar between the 2 groups (all *P* > .05) (Table 2).

**Table 2:** Number of cases and birth weight in each gestational age group of twin preterm infants in both groups (N/%, *X* ± SD, M).

Variable	Observation group (N = 72)	Control group (N = 72)	*t*/*χ*²	*P* value
Number of preterm infants (cases)			1.516	.468
27–31^+6^ W	10 (13.89%)	10 (13.89%)		
32–33^+6^ W	19 (26.39%)	13 (18.06%)		
34–36^+6^ W	43 (59.72%)	49 (68.06%)		
Birth weight (g)				
27–31^+6^ W	1374.23 ± 240.51	1280.22 ± 379.53	0.662	.510
32–33^+6^ W	1751.83 ± 272.63	1616.17 ± 404.88	1.056	.308
34–36^+6^ W	2116.75 ± 353.44	2143.38 ± 367.29	−0.354	.724

### 3.3. Comparison of congenital heart disease in twin preterm infants between the 2 groups

There were no significant differences in the incidence of patent foramen ovale (54.17% vs 61.11%, *P* = .400) and congenital heart disease (47.22% vs 36.11%, *P* = .177) between the observation and control groups. However, the incidence of atrial septal defect was significantly higher in the observation group compared to the control group (27.78% vs 13.89%, *χ*² = 4.211, *P* = .040). Other types of congenital heart disease, including patent ductus arteriosus, ventricular septal defect, and aortic stenosis, did not differ significantly between the 2 groups (all *P* > .05) (Table 3).

**Table 3:** Comparison of congenital heart disease in twin preterm infants between the 2 groups (N/%).

Variable	Observation group (N = 72)	Control group (N = 72)	*χ*²	*P* value
Patent foramen ovale	39 (54.17%)	44 (61.11%)	0.711	.400
CHD	34 (47.22%)	26 (36.11%)	1.828	.177
**ASD**	20 (27.78%)	10 (13.89%)	4.211	**.040**
PDA	15 (20.83%)	14 (19.44%)	0.043	.836
VSD	2 (2.78%)	2 (2.78%)	0.000	1.000
Aortic stenosis	1 (1.39%)	2 (2.78%)	0.340	.560

Bold values indicate statistical significance.

ASD = atrial septal defect, CHD = congenital heart disease, PDA = patent ductus arteriosus, VSD = ventricular septal defect.

### 3.4. Comparison of intracranial hemorrhage in twin preterm infants between the 2 groups

The overall incidence of intracranial hemorrhage did not differ significantly between the observation group and the control group (30.56% vs 34.72%, *P* = .596). There were no significant differences between the 2 groups in terms of the severity of intracranial hemorrhage (grades I–II vs grades III–IV) (all *P* > .05) (Table 4).

**Table 4:** Comparison of intracranial hemorrhage in twin preterm infants between the 2 groups (N/%).

Variable	Observation group (N = 72)	Control group (N = 72)	*χ*²	*P* value
Intracranial hemorrhage	22 (30.56%)	25 (34.72%)	0.284	.596
Grade I–II intracranial hemorrhage	19 (26.39%)	24 (33.33%)	0.829	.364
Grade III–IV intracranial hemorrhage	3 (4.17%)	1 (1.39%)	1.029	.310

### 3.5. Comparison of neonatal diseases in twin preterm infants between the 2 groups

The incidence of neonatal hyperbilirubinemia was significantly higher in the observation group compared to the control group (72.22% vs 55.56%, *χ*² = 4.097, *P* = .043), while the incidence of twin–twin transfusion syndrome was significantly lower in the observation group (0% vs 5.56%, *χ*² = 4.114, *P* = .042). The incidence of intracranial hypertension syndrome (thyroid hormone-binding protein) and hypocalcemia were also significantly higher in the observation group compared to the control group (9.72% vs 1.39%, *P* = .043; 12.50% vs 2.78%, *P* = .035, respectively). Other neonatal diseases such as neonatal respiratory distress syndrome, neonatal pulmonary edema, bronchopulmonary dysplasia, and neonatal pneumonia did not differ significantly between the 2 groups (*P* > .05) (Table 5).

**Table 5:** Comparison of diseases in twin preterm infants between the 2 groups (N/%).

Variable	Observation group (N = 72)	Control group (N = 72)	*χ*²	*P* value
EUGR	11 (15.28%)	14 (19.44%)	0.434	.511
ROP	1 (1.39%)	1 (1.39%)	0.000	1.000
NRDS	11 (15.28%)	10 (13.89%)	0.056	.812
Neonatal pulmonary edema	11 (15.28%)	10 (13.89%)	0.056	.812
Neonatal pneumonia	8 (11.11%)	6 (8.33%)	0.317	.575
MAS	3 (4.17%)	2 (2.78%)	0.207	.648
BPD	9 (12.50%)	11 (15.28%)	0.233	.629
Neonatal asphyxia	21 (29.17%)	20 (27.78%)	0.034	.853
Neonatal respiratory failure	2 (2.78%)	3 (4.17%)	0.207	.648
Apnea	1 (1.39%)	2 (2.78%)	0.340	.559
Pulmonary hemorrhage	0 (0.00%)	1 (1.39%)	1.007	.315
Feeding intolerance	9 (12.50%)	8 (11.11%)	0.067	.798
Gastrointestinal bleeding	0 (0.00%)	1 (1.39%)	1.007	.315
NEC	2 (2.78%)	1 (1.39%)	0.340	.559
Inguinal hernia	0 (0.00%)	2 (2.78%)	2.028	.153
Umbilical hernia	1 (1.39%)	2 (2.78%)	0.340	.559
Pulmonary hypertension	2 (2.78%)	3 (4.17%)	0.207	.648
Neonatal shock	3 (4.17%)	2 (2.78%)	0.207	.648
Myocardial injury	1 (1.39%)	2 (2.78%)	0.340	.559
Early-onset bacterial infection	3 (4.17%)	4 (5.56%)	0.150	.700
EOS	4 (5.56%)	3 (4.17%)	0.150	.700
LOS	9 (12.50%)	8 (11.11%)	0.066	.798
Purulent meningitis	1 (1.39%)	1 (1.39%)	0.000	1.000
Urinary tract infection	1 (1.39%)	0 (0.00%)	1.007	.315
Fungal infection	3 (4.17%)	4 (5.56%)	0.150	.700
**Neonatal hyperbilirubinemia**	52 (72.22%)	40 (55.56%)	4.097	**.043**
Cholestasis	4 (5.56%)	5 (6.94%)	0.118	.730
Bilirubin encephalopathy	0 (0.00%)	0 (0.00%)	0.000	1.000
Neonatal hemolytic disease	2 (2.78%)	2 (2.78%)	0.000	1.000
Preterm brain injury	3 (4.17%)	2 (2.78%)	0.207	.648
HIE	1 (1.39%)	0 (0.00%)	0.315	.648
Hydrocephalus	1 (1.39%)	0 (0.00%)	0.315	.648
**TTTS**	0 (0.00%)	4 (5.56%)	4.114	**.042**
Neonatal anemia	18 (25.00%)	20 (27.78%)	0.142	.705
DIC	1 (1.39%)	2 (2.78%)	0.340	.648
Coagulation dysfunction	17 (23.61%)	15 (20.83%)	0.160	.689
Polycythemia	3 (4.17%)	3 (4.17%)	0.000	1.000
**THOP**	7 (9.72%)	1 (1.39%)	4.764	**.043**
Low T3 syndrome	1 (1.39%)	0 (0.00%)	1.007	.315
Hypothyroidism	0 (0.00%)	1 (1.39%)	1.007	.315
Neonatal hypoglycemia	15 (20.83%)	19 (26.39%)	0.615	.431
Neonatal hyperglycemia	1 (1.39%)	1 (1.39%)	0.000	1.000
**Hypocalcemia**	9 (12.50%)	2 (2.78%)	4.822	**.035**
Hypokalemia	5 (6.94%)	5 (6.94%)	0.000	1.000
Hypoproteinemia	7 (9.72%)	5 (6.94%)	0.364	.548
Osteopathy of prematurity	0 (0.00%)	1 (1.39%)	1.007	.315

Bold values indicate statistical significance.

BPD = bronchopulmonary dysplasia, DIC = disseminated intravascular coagulation, EOS = early-onset sepsis, EUGR = extrauterine growth restriction, HIE = hypoxic-ischemic encephalopathy, LOS = late-onset sepsis, MAS = meconium aspiration syndrome, NEC = necrotizing enterocolitis, NRDS = neonatal respiratory distress syndrome, ROP = retinopathy of prematurity, THOP = thyroid hormone-binding protein, TTTS = twin-to-twin transfusion syndrome.

### 3.6. Comparison of hospitalization of twin preterm infants between the 2 groups

Regarding hospitalization, the observation group required a significantly longer duration to achieve complete enteral nutrition compared to the control group (23.78 days vs 15.88 days, *Z* = 2.397, *P* = .016). The duration of partial intravenous nutrition was also significantly longer in the observation group (22.54 days vs 11.37 days, *t* = 3.597, *P* = .027). Other hospitalization-related indicators, such as the use of noninvasive ventilation, mechanical ventilation, and the total number of hospitalization days, did not differ significantly between the 2 groups (*P* > .05) (Table 6).

**Table 6:** Comparison of hospitalization conditions of preterm infants between the 2 groups (N/%, X ± SD, M).

Variable	Observation group (N = 72)	Control group (N = 72)	*Z*/*t*/*χ*²	*P* value
Noninvasive ventilation (cases)	36 (50.00%)	34 (47.22%)	0.341	.560
Noninvasive ventilation (d)	7.91 ± 10.23	6.88 ± 7.89	0.300	.764
Mechanical ventilation (cases)	6 (8.33%)	9 (12.50%)	0.667	.414
Mechanical ventilation (d)	4.36 (1.89, 12.65)	5.53 (2.48, 8.21)	1.149	.250
PS usage (cases)	8 (11.11%)	10 (13.89%)	0.578	.447
Caffeine usage (cases)	17 (23.61%)	14 (19.44%)	0.577	.447
Total oxygen requirement (d)	12.06 ± 13.24	10.02 ± 15.78	1.101	.271
**Time to achieve full enteral nutrition (d**)	23.78 (15.23, 31.74)	15.88 (11.25, 27.93)	2.397	**.016**
**Partial parenteral nutrition duration (d**)	22.54 (14.27, 28.93)	11.37 (8.53, 19.78)	3.597	**.027**
Hospital stay (d)	21.48 ± 17.26	17.25 ± 19.68	1.502	.133
Oxygen therapy at discharge (cases)	7 (9.72%)	11 (15.28%)	0.891	.344

Bold values indicate statistical significance.

### 3.7. Comparison of perinatal conditions of mothers of twin preterm infants between the 2 groups

The age of mothers in the observation group was significantly higher than that of the control group (32.56 ± 3.26 years vs 29.56 ± 5.02 years, *P* < .001). The proportion of monochorionic twin pregnancies was significantly lower in the observation group compared to the control group (6.94% vs 54.17%, *χ*² = 26.836, *P* < .001), while the proportion of primiparous women was significantly higher in the observation group (90.28% vs 38.89%, *χ*² = 41.594, *P* < .001). The prevalence of antenatal antibiotic use (23.61% vs 11.11%, *χ*² = 3.920, *P* = .048) and hypothyroidism during pregnancy (30.56% vs 12.50%, *χ*² = 6.956, *P* = .008) were significantly higher in the observation group compared to the control group. Other perinatal maternal conditions, such as anemia and hypertension during pregnancy, did not differ significantly between the 2 groups (*P* > .05) (Table 7).

**Table 7:** Comparison of perinatal conditions of mothers of twin preterm infants between the 2 groups (N/%, *X* ± SD).

Variable	Observation group (N = 72)	Control group (N = 72)	*t*/*χ*²	*P* value
**Maternal age (yr**)	32.56 ± 3.26	29.56 ± 5.02	4.252	<**.001**
**Monochorionic twin pregnancy**	5 (6.94%)	39 (54.17%)	26.836	**<.001**
**Primipara**	65 (90.28%)	28 (38.89%)	41.594	**<.001**
Antepartum fever	5 (6.94%)	2 (2.78%)	1.352	.245
Magnesium sulfate treatment	12 (16.67%)	14 (19.44%)	0.188	.664
Corticosteroids for fetal lung maturation	40 (55.56%)	44 (61.11%)	0.456	.499
**Prenatal antibiotic use**	17 (23.61%)	8 (11.11%)	3.920	**.048**
Pregnancy complications	52 (72.22%)	41 (56.94%)	3.676	.055
Gestational hypertension	20 (27.78%)	15 (20.83%)	0.942	.331
Gestational diabetes mellitus	26 (36.11%)	18 (25.00%)	2.094	.146
Intrahepatic cholestasis of pregnancy	5 (6.94%)	2 (2.78%)	1.352	.245
Chorioamnionitis	2 (2.78%)	1 (1.39%)	0.342	.560
Anemia during pregnancy	1 (1.39%)	5 (6.94%)	2.782	.095
**Hypothyroidism during pregnancy**	22 (30.56%)	9 (12.50%)	6.956	**.008**

Bold values indicate statistical significance.

## 4. Discussion

This study employed a retrospective cohort design to compare the clinical characteristics and maternal perinatal disorders during the neonatal period between twin preterm infants conceived via IVF and those conceived naturally. The results revealed that, although there were no significant differences in basic demographic characteristics (such as gestational age, birth weight, and sex distribution) between the 2 groups, notable differences were found in the mode of delivery, incidence of premature rupture of membranes, congenital heart disease, intracranial hemorrhage, neonatal disorders, and hospitalization. Additionally, significant differences were observed in maternal age, pregnancy-related diseases, and perinatal management in the observation group.

Firstly, with respect to the mode of delivery, the proportion of assisted delivery via perineal lateral incision was significantly higher in the IVF group than in the control group. Perineal lateral incision assisted delivery was less frequent in natural pregnancies, which may reflect a preference for interventional procedures in IVF pregnancies during labor. This finding is consistent with previous studies, which have reported significant differences in the mode of delivery between IVF and naturally conceived twin pregnancies.^[13]^

Secondly, the incidence and duration of PPROM were significantly higher in the IVF group. This may be related to hormonal therapy used during IVF, the embryo transfer technique, and the underlying health status of the pregnant women.^[14]^ PPROM is a key trigger for preterm labor, and its increased incidence may elevate the risk of various complications for preterm infants.^[15]^

In terms of congenital heart disease, the incidence of atrial septal defect was significantly higher in the IVF group compared to the control group, while the incidence of patent foramen ovale and congenital heart disease did not differ significantly between the 2 groups. This suggests that IVF may be associated with an increased risk of specific congenital heart defects, warranting further investigation into the underlying mechanisms.^[16]^

Additionally, the incidence of neonatal hyperbilirubinemia, thyroid hormone-binding protein, and hypocalcemia was significantly higher in the IVF group, indicating that IVF twin preterm infants face additional health challenges during the neonatal period. These complications may be linked to maternal health during IVF pregnancies, the mode of delivery, and the intrauterine environment in which the fetus develops.^[17]^

Moreover, the IVF group required a significantly longer time to achieve complete enteral nutrition and partial intravenous nutrition during hospitalization. This reflects the greater need for nutritional support interventions in IVF twin preterm infants, likely due to higher complication rates and more complex clinical management requirements.^[18]^

Mothers in the IVF group were significantly older than those in the control group and had a higher proportion of primiparous women, which is a characteristic often seen in IVF pregnancies, as assisted reproductive technologies are more commonly used in older women.^[19]^ Furthermore, the IVF mothers had significantly higher rates of hypothyroidism during pregnancy and greater use of prenatal antibiotics compared to the control group, suggesting that these women faced more health challenges in perinatal management.^[20,21]^

The findings of this study suggest that IVF twin preterm infants face more significant health challenges during the neonatal period and require enhanced clinical management. For example, closer monitoring and timely intervention for complications such as hyperbilirubinemia, intracranial hypertension syndrome, and hypocalcemia are essential. Additionally, perinatal health management of mothers, particularly older women and those with hypothyroidism, needs to be more comprehensive, with individualized care plans tailored to their needs.

There are several limitations in this study. Firstly, as a retrospective study, the data were dependent on existing electronic medical records, which may carry the risk of incomplete or biased documentation. Secondly, the sample size was relatively small, particularly for certain complications, which may affect the reliability of the statistical analyses. Furthermore, this study focused solely on neonatal clinical characteristics and lacked long-term follow-up data to assess the developmental and health outcomes of IVF twin preterm infants. Future studies should employ a larger prospective cohort design, incorporating more IVF techniques (e.g., IVF–intracytoplasmic sperm injection, frozen embryo transfer, etc) to further explore the impact of different IVF methods on the clinical outcomes of twin preterm infants. Moreover, extending the follow-up period to assess the early childhood and long-term health of IVF twin preterm infants will provide a more comprehensive scientific basis for understanding their development.

While this study provides valuable insights into the early health outcomes of preterm twins born through IVF, it also underscores the need for future research to explore long-term follow-up data. Specifically, future studies should investigate growth trajectories, developmental milestones, neurodevelopmental outcomes, and cognitive abilities in this population. Additionally, examining the potential influence of environmental, genetic, and epigenetic factors on long-term health could provide a more comprehensive understanding of the implications of IVF in preterm twins. Such research would not only address the limitations of the current study but also contribute to the development of targeted interventions and improved clinical care for this vulnerable population.

In conclusion, this study highlights several significant differences in the neonatal and maternal perinatal health of IVF twin preterm infants, suggesting that IVF pregnancies present additional clinical management challenges while facilitating twin pregnancies. Clinicians should implement comprehensive monitoring and care for IVF twin preterm infants and their mothers, optimizing perinatal management strategies to improve both survival rates and the quality of life for these infants.

## Author contributions

**Conceptualization:** Chunyan Guo, Shasha Li, Jingcai Wang, Yanqiu Wu.

**Data curation:** Chunyan Guo, Shasha Li, Jingcai Wang, Yanqiu Wu.

**Formal analysis:** Chunyan Guo, Shasha Li, Yanqiu Wu.

**Funding acquisition:** Yanqiu Wu.

**Investigation:** Chunyan Guo, Yanqiu Wu.

**Methodology:** Chunyan Guo, Yanqiu Wu.

**Resources:** Shasha Li.

**Software:** Jingcai Wang.

**Supervision:** Shasha Li, Jingcai Wang.

**Validation:** Shasha Li.

**Visualization:** Shasha Li, Jingcai Wang.

**Writing – original draft:** Chunyan Guo, Yanqiu Wu.

**Writing – review & editing:** Chunyan Guo, Yanqiu Wu.
